# Increased expression of beta 2-microglobulin in multidrug-resistant tumour cells

**DOI:** 10.1038/sj.bjc.6600354

**Published:** 2002-06-17

**Authors:** G L Scheffer, M C de Jong, A Monks, M J Flens, C D Hose, M A Izquierdo, R H Shoemaker, R J Scheper

**Affiliations:** Department of Pathology, Free University Medical Center, Amsterdam, The Netherlands; Screening Technologies Branch, Developmental Therapeutics Program, National Cancer Institute, Frederick, Maryland, USA; Department of Oncology, Catalan Institute of Oncology, Barcelona, Spain

**Keywords:** LMR-12, multidrug resistance, expression cloning, beta 2-microglobulin, MHC class I, TAP

## Abstract

The rat monoclonal antibody LMR-12 was shown earlier to react with a plasma membrane protein, upregulated in multidrug-resistant cell lines. In this study, we observed distinct LMR-12 staining in 36 out of 55 non-drug-selected tumour cell lines, including melanomas, renal cell-, colon- and lung carcinomas, whereas in other tumour types, such as leukaemia and ovarian cancer, LMR-12 staining was generally low or absent. The cDNA encoding the LMR-12 antigen was isolated from a library of the multidrug-resistant human fibrosarcoma cell line HT1080/DR4 by expression cloning in MOP8 cells. Sequence analysis showed that the LMR-12 antigen is identical to the major histocompatibility complex class I molecule beta 2-microglobulin (β_2_-m). The LMR-12/ β_2_-m staining results were confirmed by mRNA microarray data from an independent National Cancer Institute study, as well as by newly obtained reverse transcriptase polymerase chain reaction data. Further analysis of the microarray data showed that β_2_-m levels closely reflected levels of major histocompatibility complex class I heavy chains and the transporter associated with antigen processing. Since the ABC transporter associated with antigen processing was previously shown to contribute to multidrug-resistance, it may very well be that the observed LMR-12/ β_2_-m levels are secondary to (elevated) levels of the transporter associated with antigen processing. A perspective arising from the present study is that drug resistant tumour cells may, by having elevated levels of major histocompatibility complex related molecules, be particular good candidates for alternative therapeutic therapies, such as cytotoxic T cell mediated immune-therapies.

*British Journal of Cancer* (2002) **86**, 1943–1950. doi:10.1038/sj.bjc.6600354 www.bjcancer.com

© 2002 Cancer Research UK

## 

Multidrug resistance (MDR, reviewed in Schneider *et al*, 1999) has been associated with the overexpression of genes involved in metabolism and transport of various anticancer drugs. The ‘classical’ form of MDR is associated with the product of the *MDR1* gene, P-glycoprotein (MDR1 P-gp, ABCB1), reviewed in Ambudkar *et al* (1999). MDR1 P-gp is a plasma membrane protein, that acts as an ATP-dependent efflux pump for natural product drugs. *MDR1* *P-gp* overexpression is particularly prominent in tumour cell lines selected *in vitro* by high concentrations of natural product drugs. Subsequently, in several non-P-gp MDR tumour cell lines high levels of other members of the ATP-binding cassette (ABC) transporter superfamily (Higgins, 1992) were identified: the Multidrug Resistance Protein 1 (MRP1; ABCC1; reviewed in Cole and Deeley (1998) and the Breast Cancer Resistance Protein (BCRP; ABCG2) (Doyle *et al*, 1998). Also other MRP family members (MRP2-6; ABCC2-6) (Kool *et al*, 1997; Borst *et al*, 2000) were identified to possibly contribute to MDR.

In an ongoing search for proteins involved in drug resistance we selected monoclonal antibodies (Mabs) against proteins with elevated levels in non-P-gp MDR cell lines. Immunisation of mice with the lung cancer cell line SW-1573/2R120 led to the selection of the LRP-56 Mab (Scheper *et al*, 1993). Expression cloning of the corresponding cDNA identified the M*_r_* 110 000 antigen as the human major vault protein (MVP) (Scheffer *et al*, 1995). In another endeavour using rats, we generated a panel of six Mabs reactive with proteins present at high levels in non-P-gp MDR tumour cell lines, but low or absent in the corresponding drug-sensitive, parental tumour cell lines (Flens *et al*, 1997). One of these Mabs, LMR-5, was also found to be directed to the MVP molecule, as shown by staining of MVP cDNA transfected MOP8 cells (Flens *et al*, 1997). A second one, the LMR-42 Mab, was shown to react with the endothelial cell protein C receptor (Scheffer *et al*, 2002). A third Mab in the panel, LMR-12, reacted with a plasma membrane protein of unidentified molecular weight. The protein had been found to be upregulated in several MDR cell lines (Flens *et al*, 1997) and appeared to be widely expressed in normal human tissues. Pre-labelling the tumour cells with high amounts of LMR-12 antibody was unable to increase the intracellular drug accumulation in the tumour cells (Flens *et al*, 1997). In this study we examined the presence of the LMR-12 antigen in a broad panel of drug-unselected human tumour cell lines of the US National Cancer Institute by immunohistochemical stainings. Furthermore, by expression cloning using a cDNA library of the human MDR fibrosarcoma cell line HT1080/DR4, we identified the antigen of LMR-12.

## MATERIALS AND METHODS

### Cell lines

A total of 55 cell lines of the National Cancer Institute's (NCI) anticancer drug screen panel was obtained and processed as described previously (Wu *et al*, 1992). The panel includes cell lines derived from cancers of the colon, kidney, lung, breast, ovary, and brain, as well as melanoma and leukaemia. All other cell lines used have been described in the literature and are mentioned in Scheffer *et al* (2000). The cell lines included the small cell lung carcinoma cell line GLC4 and its adriamycin-resistant (MRP1 positive) subline GLC4/ADR; the leukaemia cell lines HL60 and the adriamycin-resistant (MRP1 positive) HL60/ADR subline; the fibrosarcoma cell line HT1080 and its daunorubicin resistant subline HT1080/DR4. The polyoma transformed NIH3T3 mouse fibroblast cell line MOP8 (Muller *et al*, 1984) was used for transfection experiments.

The resistant sublines were routinely cultured in the presence of cytotoxic drug until 3–10 days before the experiments. The cell lines were cultured in RPMI-1640 or Dulbecco's modified essential medium (DMEM; Gibco Europe, Paisley, Scotland) with 10% heat-inactivated foetal calf serum (Gibco Europe) and 2 mM L-glutamine. All cell lines were passed once or twice weekly and routinely examined for *Mycoplasma* contamination.

### Tumour samples

The panel of 34 human tumours of different histogenetic origins included (snap frozen) tumours of the intestine, stomach, testis, prostate, lung, pancreas, bladder, adrenal gland, cervix, neurologic tissue, breast, ovary, kidney and melanoma.

### Immunohistochemistry

Cytocentrifuge preparations of tumour cell lines or frozen sections of tumour samples were air-dried overnight and fixed for 7 min in acetone. The slides were incubated for 1 h with LMR-12 (1 : 500) or isotype matched rat Ig in PBS containing 1% BSA. Additional control stainings were done with the W6/32 Mab. This mouse Mab is a broadly used pan-HLA class I-reactive monoclonal antibody that recognises a conformational epitope dependent on association between heavy chains and β_2_-m (Barnstable *et al*, 1978; Tran *et al*, 2001). MAb binding was detected using biotinylated rabbit anti-rat IgG (1 : 100; Dako, Copenhagen, Denmark) or anti-mouse IgG (1 : 150; Dako) and streptavidin conjugated to horseradish peroxidase (1 : 500; Zymed, San Francisco, CA, USA). Colour development was with 0.02% (w v^−1^) amino-ethyl-carbazole and 0.02% (v v^−1^) H_2_O_2_ in 0.1 M sodium acetate buffer, pH 5.0. Evaluation was done on coded slides to avoid bias in scoring. As used before for this type of analysis (Izquierdo *et al*, 1996a), a semiquantitative ‘staining index’ was calculated as the product of the fraction of positive cells and the average staining intensity estimated on a scale from 0 (negative) to 3 (very strong positive). At least three tests for each cell line were used for calculation of the average staining index. The parental GLC4 and MDR GLC4/ADR cell lines served as controls for LMR-12 staining.

### FACS analysis

One hundred μl of cell suspensions at a concentration of 1×10^6^ viable cells per ml were incubated with 10 μg LMR-12, W6/32, control Mab or rabbit polyclonal anti β_2_-m for 1 h at room temperature. Antibody binding was detected using FITC-labelled rabbit-anti-rat serum (1 : 100, Dako), -anti-mouse serum (1 : 100, Dako) or swine-anti-rabbit serum (1 : 100, Dako) for another hour at room temperature. The samples were analysed on a FACS-Star flow cytometer (Becton-Dickinson, San Jose, CA, USA).

### cDNA library and isolation of the LMR-12 cDNA clone

Previously, a cDNA-library was derived from mRNA isolated from the human MDR fibrosarcoma cell line HT1080/DR4 (Scheffer *et al*, 1995). Briefly, a size-fractionated (>2 kbp) oligo d(T) primed cDNA-library was constructed in the shuttle vector pCDM8 using non-self complementary *Bst*XI adaptors (Invitrogen, Leek, The Netherlands). Transformation of the library into the *Escherichia coli* strain MC1061/P3 by electroporation yielded approximately 100 000 primary colonies. These were divided into 10 sublibraries of 10 000 colonies each.

Bacterial subpools were grown overnight in Luria-Bertani medium, supplemented with 7.5 μg ml^−1^ tetracycline (Boehringer Mannheim B.V., Almere, The Netherlands), and 12.5 μg ml^−1^ ampicillin (Sigma Chemie, Bornem, Belgium). Plasmid DNA, containing cDNA inserts, was isolated from minipreparations of the bacterial sublibraries by alkaline lysis. MOP8 cells were transfected with isolated plasmid DNA using the DEAE-dextran method (Promega Corporation, Leiden, The Netherlands) as described by Aruffo and Seed (1987). Transfected MOP8 cells were allowed to grow for 72 h, and after trypsinization cytospins were prepared. These cytospins were air-dried overnight, fixed in 100% acetone for 7 min and stained with LMR-12 (1 : 500) to detect transiently expressed protein as described above. A colony containing the cDNA coding for the LMR-12 antigen was isolated by screening progressively smaller pools of bacterial colonies.

### cDNA sequence analysis

The LMR-12 cDNA clone was sequenced using the dideoxy Terminator Cycle Sequencing Kit on an automated 373A DNA sequencer (Applied Biosystems Benelux B.V., Maarsen, The Netherlands). Sequencing was performed in both orientations to confirm the nucleotide sequence. The data were collected and analysed using 373A computer software.

### RNA isolation and RT–PCR

Total RNA was isolated using the RNeasy 96 kit and the QIAvac vacuum manifold (Qiagen, Valencia, CA, USA) per manufacturer's protocol. Total RNA samples were quantitated using the fluorescent Ribogreen RNA quantitation reagent kit (Molecular probes) and RNA was stored at −70°C until use for RT–PCR.

The RT–PCR reactions were measured with the ABI Prism 7700 Sequence Detection System using TaqMan one-step RT–PCR SYBR green PCR master mix in 50 μl reactions using 5 ng total RNA per reaction. Primers for β_2_-m were designed with Primer Express software (PE Biosystems, Foster City, CA, USA; forward primer: GGCTATCCAGCGTACTCCAAAG, reverse primer: CAACTTCAATGTCGGATGGATG). GAPDH was used as an endogenous control (forward primer: GAAGGTGAAGGTCGGAGTC, reverse primer: GAAGATGGTGATGGGATTTC). Primer concentrations for β_2_-m and GAPDH were 600 and 100 nM respectively. Thermocycler parameters were 30 min at 48°C, 10 min at 95°C and 40 PCR cycles of 15 s at 95°C and 1 min at 60°C. All RNA samples were tested in triplicate PCR reactions. Data was analysed using the comparative CT method (Perkin Elmer, user bulletin #2) and β_2_-m was normalysed to a calibrator cell line (HCC-2998).

### Correlative and COMPARE analysis

Using SPSS 9.0 statistical software, two-tailed Pearson correlation coefficients (PCC) were calculated between (LMR-12) immunohistochemical staining data, microarray data from Ross *et al* (2000) and Scherf *et al* (2000) (available at the NCI Developmental Therapeutics Program website (http://dtp.nci.nih.gov)), and β*_2_-m* mRNA data from the RT–PCR.

The COMPARE program of the NCI Developmental Therapeutics Program was used to examine the correlation between the LMR-12 staining results and sensitivity for compounds tested, and with basal patterns of gene expression in the 55 cell lines measured by cDNA microarray.

## RESULTS

### Frequent LMR-12 staining in tumour cell lines

As reported previously (Flens *et al*, 1997), LMR-12 staining was observed in a broad panel of MDR cell lines, with higher levels in the resistant sublines. Among these cell lines are the paired parental and resistant cell lines GLC4 and GLC4/ADR, the HL60 and HL60/ADR and the HT1080 and HT1080/DR4 cell line. To further study the prevalence of the LMR-12 antigen in a panel of drug-unselected human tumour cell lines of the US National Cancer Institute's anticancer drug screening program, cytospins of these cell lines were examined for LMR-12 expression. In 36 out of 55 tumour cell lines of the panel LMR-12 staining was found (Table 1

**Table 1:**
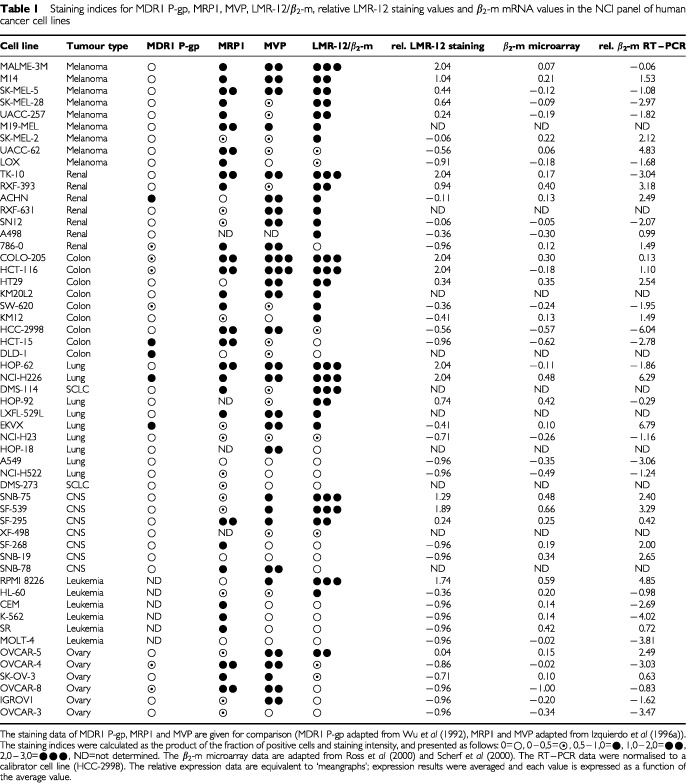
Staining indices for MDR1 P-gp, MRP1, MVP, LMR-12/β_2_-m, relative LMR-12 staining values and β_2_-m mRNA values in the NCI panel of human cancer cell lines

and Figure 1

**Figure 1:**
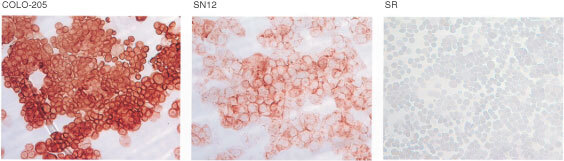
LMR-12 staining of cytospins of cell lines in the NCI cell line panel (see also Table 1); COLO-205 (colon, strongly positive), SN12 (renal, weakly positive) and SR (leukaemia, negative). Colour development was with 0.02% (w v^−1^) amino-ethyl-carbazole and 0.02% (v v^−1^) H_2_O_2_.

). In the table the staining data of MDR1 P-gp, MRP1 and MVP are given for comparison (MDR1 P-gp adapted from Wu *et al* (1992), MRP1 and MVP adapted from Izquierdo *et al* (1996a)). LMR-12 positive staining was particularly evident in distinct tumour types i.e. melanomas (seven out of nine cell lines), renal cell carcinomas (six out of seven), colon carcinomas (six out of nine cell lines) and lung carcinomas (six out of 11 cell lines). In leukemia and ovarian cancers generally low expression was observed. LMR-12 staining was frequently accompanied by MVP-, MRP1-, and/or MDR1 P-gp staining, but only with MVP staining a significant correlation was observed (*r*=0.515, *P*<0.001).

### β_2_-m is the LMR-12 antigen

As reported previously (Flens *et al*, 1997), Western blot experiments did not give information on the nature of the cognate antigen of the LMR-12 Mab, not even when ^125^Iodine-labelled LMR-12 was used in the experiments. Also, immunoprecipitation experiments did not reveal the antigen. Therefore, the expression cloning system that had succesfully been used to identify the nature of the LRP-56 Mab (Scheffer *et al*, 1995) was also used here. MOP8 cells were transiently transfected with pCDM8 vector, containing cDNA inserts of the MDR fibrosarcoma cell line HT1080/DR4 and screened for LMR-12 immunoreactivity. In the primary screening, MOP8 cells transfected with cDNAs from one of the sublibraries showed one single LMR-12 immunoreactive MOP8 cell among approximately 30×10^6^ cells. The coding cDNA clone was isolated from the sublibrary after several cycles of screening of MOP8 cells transfected with cDNA's from progressively smaller pools of bacterial colonies for transiently expressed antigen.

Sequence analysis of both ends of the LMR-12 cDNA, and database searches with the obtained sequence information revealed that the LMR-12 antigen is the human major histocompatability complex (MHC) protein β_2_-m.

### LMR-12/β_2_-m staining data correlates with mRNA data

To confirm the expression data on β_2_-m in the cell line panel, the correlation between the LMR-12/ β_2_-m staining data and microarray data on mRNA levels for β*_2_-m* was examined. Ross *et al* (2000) and Scherf *et al* (2000) have reported microarray data on approximately 8000 genes in the cell lines of the NCI panel, including β*_2_-m*. These data are available via http://dtp.nci.nih.gov. From a total of 45 cell lines, both protein and mRNA data could be compared. The analysis showed a moderate, but highly significant correlation (Tables 1 and 2

**Table 2:**
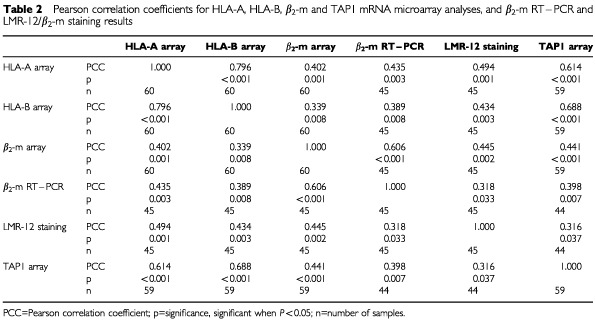
Pearson correlation coefficients for HLA-A, HLA-B, β_2_-m and TAP1 mRNA microarray analyses, and β_2_-m RT–PCR and LMR-12/β_2_-m staining results

and Figure 2

**Figure 2:**
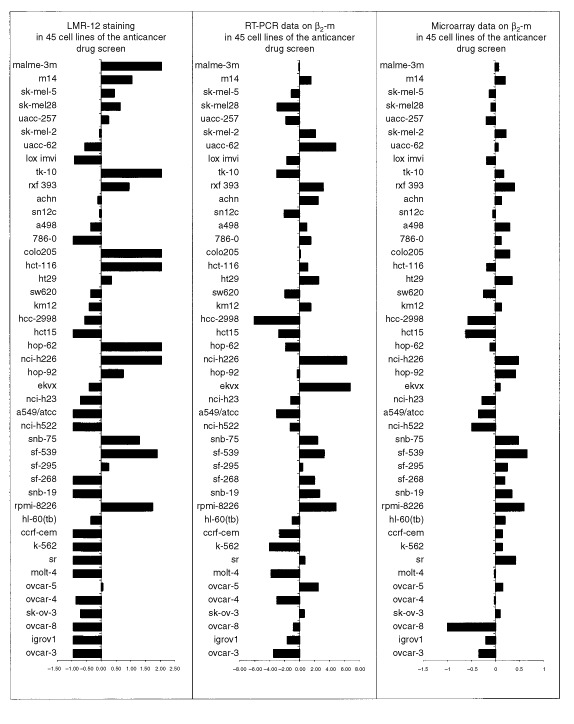
Meangraphs of LMR-12 staining, β_2_-m RT–PCR data and β_2_-m microarray data in 45 cell lines of the anticancer drug screen. The RT–PCR data were normalised to a calibrator cell line (HCC-2998). The β_2_-m microarray data are available at http://dtp.nci.nih.gov. The meangraphs were made from relative expression data; expression results were averaged and each value was expressed as a function of the average value (see Table 1). Correlations calculated from these data are presented in Table 2.

) for β_2_-m protein and mRNA data in these studies.

To further explore the correlation between LMR-12/ β_2_-m protein and mRNA data, a light-cycle RT–PCR was performed on RNA isolated from the NCI cell lines. These RT–PCR values of the cell lines, normalised to a calibrator cell line (HCC-2998), also showed a moderate, but significant correlation with the LMR-12/ β_2_-m staining data (Tables 1 and 2 and Figure 2). Correlation between RT–PCR data and microarray data was highly significant (Tables 1 and 2 and Figure 2).

To further examine the correlation of the LMR-12/ β_2_-m data with MHC class I associated molecules, statistical analyses were done on microarray data available for these molecules. (Highly) significant correlations were observed for both LMR-12/ β_2_-m staining data and β*_2_-m* mRNA data with microarray data for the MHC class I heavy chains HLA-A and HLA-B, as well as with the ABC transporter associated with antigen processing TAP1 (Table 2).

### LMR-12/β_2_-m staining correlates with W6/32 MHC class I staining

**Figure 3:**
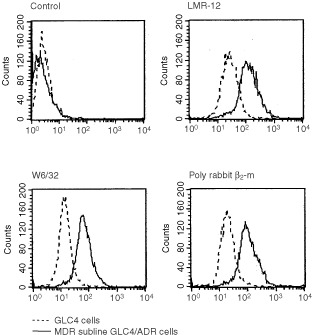
FACS analysis of GLC4 and MDR subline GLC4/ADR cells with control Mab, LMR-12, W6/32 and rabbit polyclonal anti β_2_-m. The different anti-MHC class I anti sera show similarly positive signals on the GLC4 cells, which are equally increased on the MDR subline GLC4/ADR. Antibody binding was detected using FITC-labelled rabbit-anti-rat serum, -anti-mouse serum or swine-anti-rabbit serum. The samples were analysed on a FACS-Star flow cytometer.

To further confirm the association of LMR-12/ β_2_-m staining with MHC class I heavy chain, we FACS analysed the staining of LMR-12/ β_2_-m, the anti-MHC class I Mab W6/32 and a polyclonal anti β_2_-m antiserum in paired parental and MDR cell lines. The results observed with the latter two reagents were highly comparable to the LMR-12 results. Elevated staining of all three antisera was observed in the GLC4/ADR cells and in the HL60/ADR cells compared to their parental cell lines (Figure 3

).

**Figure 4:**
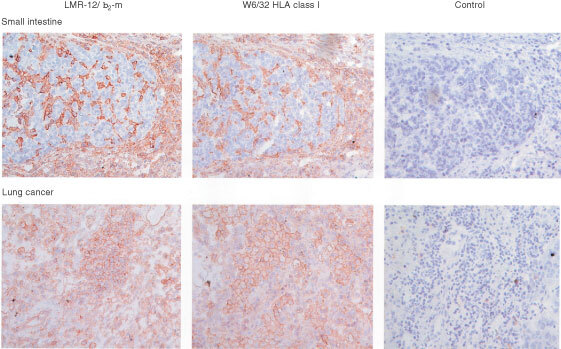
Staining of frozen sections of tumour samples with LMR-12, W6/32 and control Mab. Upper panel: small intestinal cancer samples (showing only staining in the surrounding tissue). Lower panel: lung cancer samples (showing staining in the tumour cells and in the surrounding tissue). In the two panels a similar staining pattern is observed for both LMR-12/ β_2_-m and W6/32 HLA class I molecules. Colour development was with 0.02% (w v^−1^) amino-ethyl-carbazole and 0.02% (v v^−1^) H_2_O_2_.

In a panel of 34 human tumours of different histogenetic origin, clear LMR-12/ β_2_-m staining was observed in ∼90% of the tumour samples, while the surrounding normal tissue always stained positive. The staining pattern of LMR-12/ β_2_-m was closely paralleled by W6/32 staining (Figure 4

).

### COMPARE analysis

When the LMR-12/ β_2_-m staining values for the tumour cell lines were utilised as a ‘seed’ pattern for the COMPARE program, no correlations greater than 0.46 were observed for anticancer agents tested. Extensions of these analyses to the whole synthetic database showed no correlations greater than 0.6 and no clear chemical structural theme could be observed in the top ranked compounds. Application of the COMPARE analysis to the molecular targets database yielded MVP as the second ranked pattern. The first ranked pattern was the BCL2-antagonist of cell death (BAD). While these were the best correlations observed, they were not particularly strong, with Pearson correlation coefficients (PCC) in the 0.3–0.4 range. MRP1 also showed up in this analysis, ranked at position 17 with a PCC of 0.2. The top reverse COMPARE hit was glutathione S-transferase (GST) pi with a PCC of 0.454.

Comparison to the microarray database yielded several unfamiliar genes in the top 10, but also two MHC class I molecules, F and A, at position 11 and 12, respectively, and with PCCs of around 0.5. Also, β_2_-m showed up in the analysis, at position 26, with a PCC of 0.47.

## DISCUSSION

Staining with the rat antibody LMR-12 was reported to be upregulated in a broad panel of MDR cell lines (Flens *et al*, 1997). In this study we found that the LMR-12 antigen is also present in 36 out of 55 samples of a panel of drug-unselected primary tumour cells. Staining was particularly evident in distinct tumour cell types that are in general considered as refractory to chemotherapy, i.e. melanomas, renal cell-, colon- and lung carcinomas. Tumour cells considered more sensitive to chemotherapy, such as leukaemia and ovarian carcinoma showed only low staining levels. LMR-12 staining was never exclusive and correlated significantly with MVP staining, but not with MRP1 or MDR1 P-gp staining. Collectively, these results suggested the potential relevance of the cognate antigen in resistance of tumour cells to cytostatic drug exposure. Considering that drug resistance continues to represent a major obstacle in clinical cancer treatments, we set out to identify the antigen recognised by the LMR-12 Mab. As conventional Western blot and immunoprecipitation techniques had proven unsuccesful to give information on the identity of the antigen of this Mab, we used an eukaryotic expression cloning procedure. The LMR-12 MAb was used as a probe to detect transiently expressed protein in transfected MOP8 cells and to isolate the cDNA encoding the antigen.

Sequence analysis and database searches showed that the LMR-12 antigen has no homology with ABC transporter proteins involved in MDR. Instead, the protein turned out to be identical to the human MHC class I molecule β_2_-m. The M_*r*_ 12 000 β_2_-m associates with MHC class I heavy chains to form class I human leukocyte antigen (HLA) complexes.

When comparing our LMR-12/ β_2_-m staining data in the NCI cell line panel to independent, previously reported β*_2_-m* mRNA microarray data and to newly obtained RT–PCR mRNA data, we observed moderate, but significant correlations between protein and mRNA data. The RT–PCR data correlated highly significantly with the microarray data. As yet, only a few reports have been published on the correlation between mRNA and protein levels in cells and tissues, whereas none has correlated microarray data with RT–PCR data. Similar to our observations, Anderson and Seilhamer (1997) found a correlation coefficient of 0.48 for mRNA levels and proteins expressed in human liver. These, acceptable, but non-perfect correlations indicate that post-transcriptional regulation of gene expression is a frequent phenomenon in higher organisms and very likely holds true for β_2_-m as well. Furthermore, β_2_-m transcript levels appeared to vary considerably in individual cell lines, making β_2_-m a poor candidate for standardising mRNA levels as detected by e.g. RT–PCR. Although similar findings were already reported by Finnegan *et al* (1993), still many researchers erroneously choose β*_2_-m* as a standard housekeeping gene.

The observed significant correlation between microarray data and protein data as well as mRNA data obtained by RT–PCR, indicates that publicly accessible databases containing microarray data for large numbers of genes can be an important valid information source for researchers.

Further accession of the microarray database to correlate our β_2_-m results to reported microarray data on MHC related molecules showed that both β_2_-m staining and mRNA data (highly) significantly correlated with mRNA data for MHC class I heavy chains and TAP1. The connection of the LMR-12/ β_2_-m data with MHC class I heavy chains was also confirmed by staining results with the W6/32 Mab both in FACS experiments and immunohistochemical stainings of human tumour sections. Also in the COMPARE analysis to the microarray database MHC class I molecules turned up at high positions in the list. When compared to molecular targets, interesting molecules such as GST pi, BAD and the MDR marker MVP turned up, but no high correlations were observed for anticancer agents tested.

As β_2_-m itself has no characteristics of a transporter protein, it seems very unlikely that the protein itself plays a causal role in the MDR phenotype. Still, upregulation of MHC related molecules in MDR cells was already observed in previous studies. Iguchi *et al* (2001) noted upregulation of MHC class I molecules, including β_2_-m, in gastric- and colon cancer cell lines after treatment with the plant polysaccharide PSK, which was examined for anti-tumour effects. Furthermore, in a study to examine the possible contribution of the ABC transporter associated with antigen processing (TAP) to MDR, we showed that in our panel of MDR cell lines elevated TAP levels were paralleled by elevated levels of MHC class I molecules (Izquierdo *et al*, 1996b). The TAP molecule plays a major role in MHC class I-restricted antigen presentation by mediating peptide translocation over the endoplasmic reticulum membrane. In the same study a moderate contribution of TAP to MDR was demonstrated by transfection of the TAP genes into TAP-deficient lymphoblastoid T2 cells, resulting in low level resistance to etoposide, vincristine and doxorubicin. Therefore, it seems valid to conclude that the observed elevated levels of β_2_-m in this study most likely are the consequence of upregulation of TAP. Recently, the antigen of LMR-42, another MDR-related Mab originating from the same study as the LMR-12 Mab (Flens *et al*, 1997), was identified as the endothelial cell protein C receptor (EPCR). This type 1 transmembrane glycoprotein is also a member of the MHC superfamily (Scheffer *et al*, 2002). In contrast to these findings of upregulated MHC related molecules, many studies have reported on downregulation or loss of expression of MHC class I related molecules in malignant cells (Kaklamanis *et al*, 1995; Korkolopoulou *et al*, 1996; Keskinen *et al*, 1997; Gudmundsdottir *et al*, 2000; Kamarashev *et al*, 2001; Palmisano *et al*, 2001). Ogretmen *et al* (1998) even reported the loss or decreased expression of β_2_-m in drug-resistant sublines of MCF-7 and HL60 cells, controlled by post-transcriptional mechanisms. These authors also reported that the partial inhibition of β_2_-m by antisense RNA resulted in 2–3-fold decreased sensitivity of MCF-7 cells to doxorubicin and mAMSA. An explanation for these opposing results may be, partially, found in utilising other (sub) lines of cell lines and differences in culture conditions. MHC class I levels may be influenced by differences in culture medium components, and the presence or absence of stimulating cytokines such as interferon gamma (Keskinen *et al*, 1997). From a different perspective the obtained results may be interesting for therapeutic treatments as well. Drug resistant tumour cells may, by having elevated levels of MHC related molecules, be particular good candidates for alternative therapies, such as cytotoxic T cell mediated immune-therapies.
